# Editorial Notes

**Published:** 1912-12

**Authors:** 


### Editorial Notes.

---

**Dr. Robert Fletcher.** THE death of Dr. Robert Fletcher deprives the Bristol Medical School and Royal Infirmary of their oldest student, and the world of medicine of its bibliographer-in-chief. By this sad circumstance a contribution by Dr. Fletcher to this *Journal* appears side by side with the intimation of his decease, while an appreciative article which his friend Sir William Osler had promised for publication in this issue has been changed by the melancholy event into an obituary notice.

Dr. Fletcher to the last was keenly interested in his native city and his old Medical School, now turned University. During the past summer he had his photograph specially taken so that he might present a copy to the Bristol Medico-Chirurgical Society, and no apologies are necessary for publishing the letters to the Secretary with which he accompanied the gift. The first letter was in response to a request that the Society might have a copy of his portrait to add to its collection.

*June 22nd,* 1912.

MY DEAR SIR,

Your courteous letter of 3rd inst. was duly received, and gave me much pleasure. I have delayed answering it, endeavouring to find a photograph of myself, which you have so kindly proposed to hang on the walls of the Society's rooms. I have not been successful in this, and I have desired a fresh photograph to be made, which I will send you as soon as received. I may add that some three of four years ago I received a gold medal of the Royal College of Surgeons of England in acknowledgment of the work done to which you refer.

I have always treasured my recollections of the Bristol Medical School and Infirmary with much pleasure and interest. I was articled to Surgeon Henry Clark for the usual five years, the last two of which I spent at the London Hospital, being a pupil of Surgeon James Luke, and of a physician on the medical side whose name I have forgotten.

I resigned my editorship of the *Index Medicus* because the work was mostly done at night, and in my advanced age I found it too trying for my sight. I still, however, retain my connection with the Library of the Surgeon-General's Office.

Thanking you again for your kind letter,

I remain,

Very truly yours,

ROBERT FLETCHER.

The second letter announces the dispatch of the portrait.

*July* 10*th*, 1912.

MY DEAR SIR,

The photographer has kept me waiting a long time for the promised copy of my likeness. It was sent off to-day by registered mail, and I hope will reach you uninjured. My friends consider it to be an excellent likeness.

Believe me to be,

Very sincerely yours,

ROBERT FLETCHER.

The third was written in answer to the acknowledgment of the safe arrival of the portrait. In this reply opportunity had been taken of congratulating Dr. Fletcher on the honorary degree of M.D. which had been offered him by the University of Bristol. This distinction was conferred upon him *in absentia* by the Chancellor of the University at his Installation on October 17th, and he appreciated the honour highly.

*July* 29*th*, 1912.

DEAR SIR,

Permit me to thank you for your letter of the 19th inst. I received the invitation from the University to be present at the Installation of the Chancellor, and to receive the honorary degree of M.D., but was obliged to announce the impossibility of my being present. It would give me very great pleasure to receive in person the degree from the University of my native city, but I fear to risk two ocean voyages in the autumn.

With kindest wishes, believe me to be,

Very truly yours,

ROBERT FLETCHER.

Of the magnitude of Dr. Fletcher's life-work, and its inestimable value in advancing medical science, Sir William Osler speaks elsewhere. Our task is to offer to Dr. Fletcher's family and relatives, both in America and England, our deepest sympathy with them in their loss, a loss in which we humbly claim to share, though not without a tinge of pride that our city and Medical School should have bred so fine an instance of what may be achieved "within the hour-glass of one man's life."

**Resignation of the Editor.**

WITH the completion of Volume XXX. of this *Journal* the Editor resigns the pleasurable duty which he has had the honour to carry on for twenty-one years, since Mr. Greig Smith resigned it after the first nine years of the *Journal's* life.

The *Journal* Committee cannot be otherwise than gratified that their efforts have been so well supported, that the *Journal* has been an unqualified success, for it obviously has been a success, and not only in its literary capacity, but also from its very important work of building up and maintaining the Library.

The retiring Editor trusts that the *esprit de corps* which has always existed since its foundation may long be continued, and that the Committee may continue to have the whole-hearted support of the Society in the editorial work, which does honour to themselves and the University with which the Society is associated.

